# Resilience-Focused HIV Care to Promote Psychological Well-Being During COVID-19 and Other Catastrophes

**DOI:** 10.3389/fpubh.2021.705573

**Published:** 2021-08-04

**Authors:** L. Lauren Brown, Erika G. Martin, Hannah K. Knudsen, Heather J. Gotham, Bryan R. Garner

**Affiliations:** ^1^Department of Psychiatry and Behavioral Sciences, School of Medicine, Meharry Medical College, Nashville, TN, United States; ^2^Infectious Disease Division, Department of Medicine, Vanderbilt University Medical Center, Nashville, TN, United States; ^3^Department of Public Administration and Policy, Rockefeller College of Public Affairs and Policy, University at Albany, Albany, NY, United States; ^4^Center for Collaborative HIV Research in Practice and Policy, School of Public Health, University at Albany, Albany, NY, United States; ^5^Department of Behavioral Science, College of Medicine, University of Kentucky, Lexington, KY, United States; ^6^Department of Psychiatry and Behavioral Sciences, Stanford University School of Medicine, Palo Alto, CA, United States; ^7^Community Health and Implementation Research Program, RTI International, Durham, NC, United States

**Keywords:** resilience, COVID-19, HIV, resilience-focused HIV care, behavioral health, peer support

## Abstract

The COVID-19 pandemic has adversely affected people with HIV due to disruptions in prevention and care services, economic impacts, and social isolation. These stressors have contributed to worse physical health, HIV treatment outcomes, and psychological wellness. Psychological sequelae associated with COVID-19 threaten the overall well-being of people with HIV and efforts to end the HIV epidemic. Resilience is a known mediator of health disparities and can improve psychological wellness and behavioral health outcomes along the HIV Continuum of Care. Though resilience is often organically developed in individuals as a result of overcoming adversity, it may be fostered through multi-level internal and external resourcing (at psychological, interpersonal, spiritual, and community/neighborhood levels). In this *Perspective*, resilience-focused HIV care is defined as a model of care in which providers promote optimum health for people with HIV by facilitating multi-level resourcing to buffer the effects of adversity and foster well-being. Adoption of resilience-focused HIV care may help providers better promote well-being among people living with HIV during this time of increased psychological stress and help prepare systems of care for future catastrophes. Informed by the literature, we constructed a set of core principles and considerations for successful adoption and sustainability of resilience-focused HIV care. Our definition of resilience-focused HIV care marks a novel contribution to the knowledge base and responds to the call for a multidimensional definition of resilience as part of HIV research.

## Introduction

The COVID-19 pandemic poses a substantial threat to national and jurisdictional efforts to end the HIV epidemic (1). In the United States (U.S.), a country disproportionately affected by COVID-19, the pandemic has been connected with losses to care and viral failure among people with HIV (2). Pandemics, other catastrophes, and adverse events are also known to exacerbate pre-existing substance use disorders (SUDs) and other behavioral health outcomes and increase the likelihood for posttraumatic stress disorder (3–5). Worsening behavioral health conditions in turn adversely affect all aspects of the HIV Continuum of Care (5), including HIV transmission and disease progression (6–8).

The protracted nature of the COVID-19 pandemic has created new and overwhelming health challenges for the general public, including people with HIV. Over half (53%) of the general population report that COVID-19 has negatively affected their mental health (9). People with HIV experience higher rates of stress and psychological trauma than persons who are HIV-negative (6–8) and consequently are at heightened risk for deleterious health outcomes resulting from the pandemic. Thus, there is a critical need for HIV care providers to address psychological wellness among people with HIV.

Decades of research highlight *resilience*, or the human capacity for overcoming adversity, as a key factor in determining psychological wellness (10, 11). Global research has documented how resilience is negatively associated with depression, anxiety, and behavioral problems, but positively associated with psychological well-being and quality of life (12–17). More recently, resilience has been identified as a mediator of health disparities (18) and a mechanism for improving HIV outcomes (19, 20). Although resilience can play a critical role in buffering the negative effects of disasters and catastrophes such as the COVID-19 pandemic (11), the role of resilience in HIV care has not been fully studied, and a model of resilience-focused HIV care needs operationalization. Given the connection between resilience and improved psychosocial and HIV outcomes, we posit that adoption of a resilience framework during the COVID-19 pandemic may assist HIV care providers in promoting salutogenesis – a focus on the factors that foster well-being and contrasts a more traditional pathogenic focus on the factors that contribute to risks and adverse health outcomes (21). In this *Perspective*, we outline core principles of resilience-focused HIV care, offer recommendations for initiating its adoption, and illustrate the potential impact of implementing these recommendations on the HIV Continuum of Care. Ideally, future research may build on the principles and recommendations we provide in this *Perspective*, as well as assess the effectiveness of different strategies to foster resilience in different populations.

## Resilience-Focused HIV Care

The Federal Emergency Management Administration defines resilience as a culture of preparedness that is fostered through programs that mitigate harm (22). In medical care, the concept of resilient health care refers to a clinic, hospital, or care organization learning from adverse events (i.e., learning how harms could have been prevented or minimized if there had been greater responsiveness) and adapting clinic procedures to reduce future hazards or harm (23). Efforts to promote resilient systems may perhaps be viewed as critical components to averting acts of misfeasance as part of “*do no harm*.” Organizations or institutions that are “trauma resilient” have adopted the principles of trauma-informed care and have done so through a resilience lens to cultivate the safe, stable, and nurturing environment necessary for reducing adversity and promoting growth.^1^ In a trauma-resilient system, the impacts of adversity on patients and providers are considered and procedures are adapted to reduce negative effects. For example, all patients have access to trauma and resilience screenings, with services adapted to meet patient needs (e.g., procedures are communicated in a way that is sensitive to patients' experiences or those who might be overwhelmed), and workforce procedures are adjusted to reduce the likelihood that providers will experience vicarious trauma (e.g., effects of hearing and holding patients' trauma are discussed as part of regularly scheduled supervision) (24).

Resilience may be more broadly defined as positive adaptation to adversity, with the conditions in which adversity arise being critical in determining resiliency (10). Psychologically, humans are primed for recovery because the brain is malleable and may be rewired (neuroplasticity). While resilience can develop as one gains a sense of mastery over adversity, some individuals experience more pervasive trauma effects leading to imprinting in the brain; this may require external forces or interventions to open “windows of plasticity” to establish new neural pathways associated with positive adaptation (25). Thus, although resilience often develops organically, it may also be fostered through internal and external resourcing, as individuals are provided with the necessary skills and supports (structural or interpersonal) to overcome stressors. For example, for people living with HIV, stigma, poor family support, human rights abuse, and insecurities (e.g., housing and food insecurity) impose a “load” of stressors that may inhibit a person's ability to positively adapt without additional resources. Conversely, positive intimate partnerships, supportive HIV serostatus disclosure experiences, and awareness of legal protections for people with HIV may promote resilience (26). It is notable that factors affecting resilience in people with HIV vary across cultures and countries (26–28).

**Figure 1:**
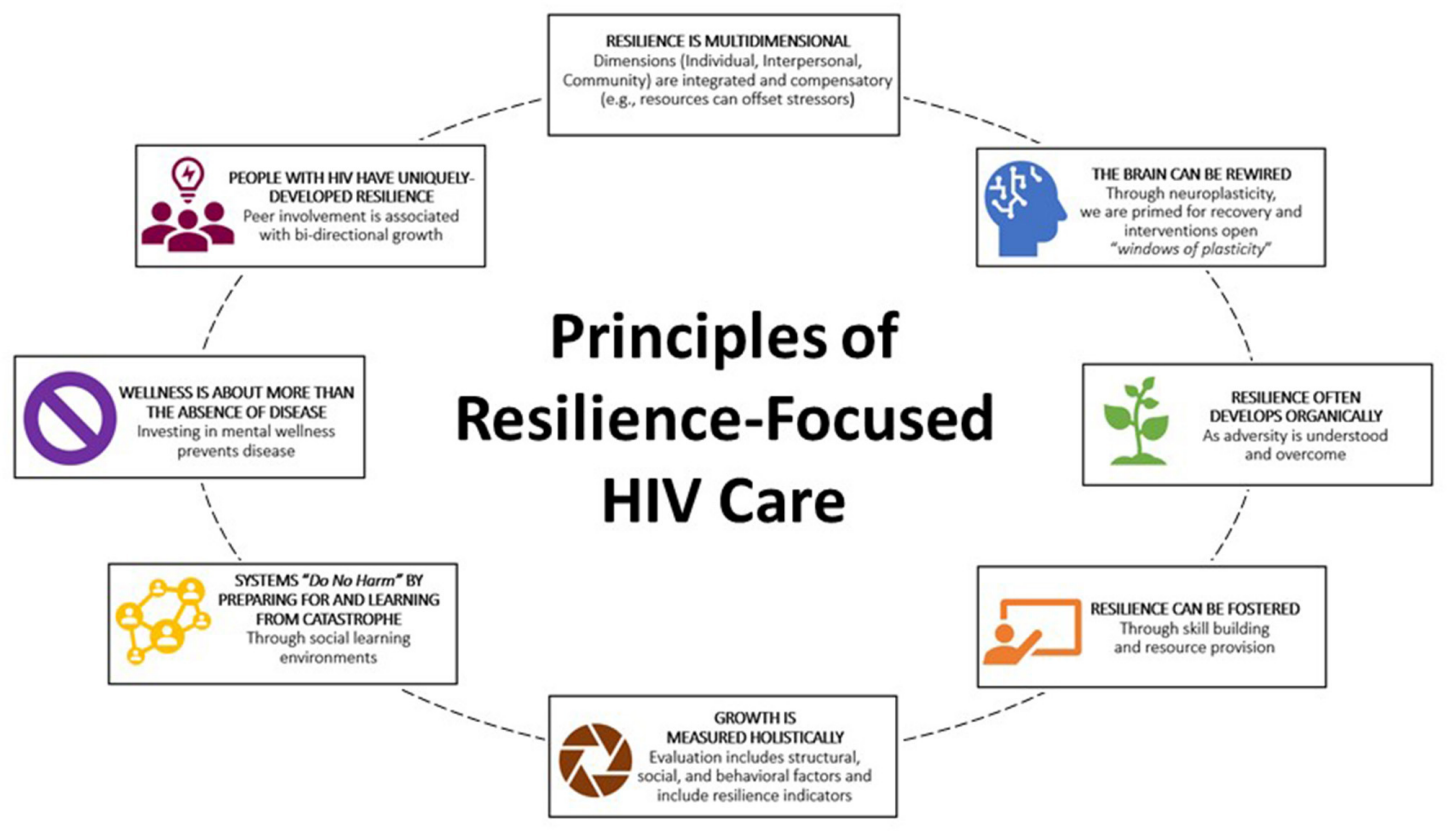
Principles of resilience-focused HIV care. Source: Authors' synthesis of the literature.

We describe resilience-focused HIV care as a care model to help providers promote optimum health for people with HIV by providing multi-level resourcing to buffer the effects of adversity and foster overall well-being consistent with a salutogenic approach (see Figure 1). A narrative review of relevant literature (19, 20) suggests that resilience-focused HIV care differs from standard HIV care in that it directs practitioners to improve the HIV Continuum of Care by helping individuals develop resources in multiple dimensions of their socio-ecological context, rather than generally directing efforts predominately at the individual level. Resourcing is sought at each contextual level as a multifaceted mechanism for adapting to and overcoming stressors and buffering social determinants of health (e.g., gender inequality, poverty). These dimensions include: internal (psychological strength; self-awareness and self-care; hopefulness about life and future); interpersonal (HIV-related facilities, social supports from family); and community (religion and spirituality, social support from community), with overlap and some interdependence between the levels.

The adoption and implementation of resilience-focused HIV care may help providers understand the unique adversities faced by people with HIV, the impact of adversity on improving outcomes along the HIV Continuum of Care, and how the psychological strengths of those who have overcome adversity can be leveraged for effective HIV care planning, especially during the COVID-19 pandemic and other catastrophes. This resilience framework breaks from a traditional medical model, which has been criticized for applying a pathologizing lens that views individuals as ailing and requiring “expert” treatment in order to be healed (29); it may help providers experientially understand (through increased emotional intelligence) the unique strengths and mastery that people with HIV have developed as a result of major life adversity. This improved experiential understanding may help providers learn strategies for promoting resourcing that can improve wellness in people with HIV.

By adapting some of the assumptions of trauma-informed care (30), we recommend HIV providers situate HIV care within a resilience framework through a process of *realizing* (adversity impacts health but may be mitigated through effective resourcing), *recognizing* (learning to understand and identify signs of adversity and resilience), and *responding* (developing and implementing resilience-focused services) (30). We provide seven core principles of resilience-focused HIV care and offer five recommendations for how systems of HIV care may begin the process of adopting and implementing resilience-focused HIV care to systemically respond to and prepare for catastrophes. These recommendations have been informed by existing theories and our experiential understanding of organizational-level change in HIV care settings. Theories informing our recommendations include trauma-informed care (19), organizational resilience (10), organizational trauma resilience (16), and multidimensional resilience in HIV care (12). Although more research is needed to define the core constructs of resilience-focused HIV care as a multi-level intervention, we hope these principles and recommendations can be leveraged in the development and evaluation of future resilience-focused HIV care interventions.

## Recommendations for Adopting Resilience-Focused HIV Care

### Increase Providers' Awareness of the Role of Resilience

Champions of resilience-focused HIV care should seek to educate providers within their system by increasing awareness of the role of resilience. Ongoing learning objectives might include: (1) Define resilience, explaining it is not a pre-ordained or exceptional personality trait but rather the effects of internal or external resourcing that can be developed after adapting to major adversity, emotional pain and stress, and particularly as one gains a sense of mastery from successfully overcoming barriers or utilizing external resourcing (e.g., structural support, social support) to offset stressors (28, 31) (2) Increase provider realization that resilience (a) mediates health outcomes, (b) is prevalent among people with HIV, and (c) may be developed; and (3) Learn how resilience has played an important role in the health outcomes of people with HIV by illustrating germane resilience attributes—such as self-efficacy, patient activation, optimism, and conscientiousness—that have been connected with improvements in antiretroviral therapy adherence, CD4 cell count, and viral suppression (19). Further information about the role of resilience in mediating HIV outcomes may be found in a recent systematic review (19).

### Integrate Resilience Screening as Part of HIV Clinical Care

After learning about the role and importance of resilience, providers must learn to recognize resilience among people with HIV, which can be done through implementation of screening processes. Validated resilience screening tools (32–34) can be used to measure the psychological components of the construct. Although there is a need for the development of instruments to comprehensively measure dimensions of resilience along the socio-ecological model of health, the Resilience Scale for Adults assists with conducting unidimensional measurements of psychological- and interpersonal- levels (34). Organizations providing direct care may consider using this instrument with additional items to measure community, neighborhood, and societal-level dimensions of resilience (e.g., community resources including the patient's ability to take advantage of these resources, neighborhood access, legal or cultural protections) (26). During this point in time, this should include COVID-19 specific stressors such as loss of job, housing insecurity, and isolation. However, adding screening to HIV clinical visits is just the first step in this process. Care organizations also should implement clinic protocols to interpret the screening tools, meet with patients to review the results and jointly develop a resourcing plan, and also provide referrals to and ease access to community social service providers.

### Include Resilience Indicators as Part of Continuous Quality Improvement

Results from resilience screenings should be included as part of continuous quality improvement (CQI), so that providers may gauge if there are any positive associations between programs and resilience indicators. Subsequently, as associations are observed, evaluators should share findings with stakeholders to improve awareness within the system (patient and provider) and with those invested in the system (cross-sector collaborators). Findings should be shared in public forums (e.g., town halls) to allow stakeholders to provide input to address outcomes negatively associated with resilience indicators.

### Enhance the Integration of Behavioral Health Services Into HIV Clinical Care

Responsive relationships are a type of human resource known to offset adversity and promote interpersonal-level resilience. Behavioral health services, especially in light of COVID-19 related increases in mental health and substance use issues (23), are one type of responsive relationship that could be offered in settings that have not traditionally provided this care, as well as be provided as on-site services or offsite collaborations with HIV Service Organizations (HSOs). For HIV primary care centers with the established infrastructure to expand services, the American Medical Association offers open-access modules with strategies and necessary steps for integrating behavioral health into primary care (35). Should primary care providers not have the capacity, services may still be prioritized and promoted through warm handoffs and on-going engagement with HSOs offering standard behavioral health support.

### Facilitate Peer Support

Peer programs are common in HSOs, and should also be prioritized as a primary type of psychological support and delivered through a resilience-framework. Peer supports are services provided by persons with HIV and have been shown to be mutually beneficial in that both patients and providers experience positive effects (36). For peers providing the care, many experience post-traumatic growth or positive impacts following traumatic experiences and/or reductions in depressive symptoms (37). Though HIV diagnosis itself is often proceeded by a period of denial or struggle, many individuals ultimately experience something called Identity Reformation, where a new identity is built and “in which successfully living with HIV is a central positive element” and connected with a new-found ability to help other people living with HIV [(pg. 7)] (38). Individuals receiving peer support experience enhanced social attachment and reassurance of worth, improvements in attitudes and cognitions, increased HIV knowledge, decreased sexual-risk taking behaviors and substance use, and greater engagement with neighborhood and community resources—all of which are associated with improved health in people with HIV (19, 39–41). Given the known negative impact of COVID-19 on the physical and mental health of people with HIV (decreased medication adherence, increased social isolation, and worsened mental health), expanding peer support may be a vital resource for individuals who might otherwise be invisibly struggling. Resilience-focused peer support could entail tracking peer resilience indicators to observe if there is also a positive association between peer involvement and their own continued resilience, with institutional changes in response to peer needs.

## Considerations for Successful Implementation and Sustainment of Resilience-Focused HIV Care

Successful implementation and sustainment of resilience-focused HIV care hinge on several important factors, including adequate funding, leadership support, and on-going CQI. To sufficiently *scale out* (adapt this evidence-based intervention to a new population or delivery system) and *scale up* (implement this evidence-based intervention across a health system) (42) key aspects of resilience-focused HIV care (such as the provision of responsive relationships), HIV funders must prioritize behavioral health services and peer support by committing funding to expand and enhance these programs. HIV Service Organizations offering behavioral support have likely experienced an increased demand for services as a result of COVID-19. As such, many may require increased funding to effectively respond to increased need. More specifically, earmarked funding is needed to respond to the increased need for trauma-informed substance use and mental health services. Concurrently, institutional leadership must prioritize this model of care to ensure systemic adoption and commitment through on-going workforce development, CQI, and continual adaptation of service provision in response to CQI findings.

## Potential Application and Impact of Resilience-Focused HIV Care Model

In addition to increased retention in care and adherence to medication, resilience-focused HIV care has the potential to mitigate numerous stressors, including related to COVID-19 (43). Pandemic-related stressors impacting people with HIV in the US include loss of job, childcare, other financial resources, and housing (44). Workers in healthcare settings have also faced major challenges, with significant increases in stress and anxiety (45). We hypothesize that, when adopted systemically, both patients and HIV care providers may benefit from the adoption of resilience-focused HIV care. Specifically, patients grappling with resource needs would benefit from the assessment and provision of multidimensional resilience resourcing. Peers may be best suited for assessing comprehensive needs spanning financial struggles (housing, childcare, etc.) to stigma and self-efficacy. After assessing these needs in multiple dimensions, patients and providers may employ comprehensive care planning, leading to more powerful health effects as goals are achieved across multiple domains. Additionally, providers who see their patients achieve goals are less likely to experience compassion fatigue and more likely to experience energy-enhancing compassion satisfaction that is associated with better professional quality of life (46).

## Discussion

In light of the myriad stressors connected to COVID-19, HIV providers have been tasked with modifying standard HIV care to ensure people continue to be retained in care and adherent to medication regimens. The established relationship between psychological stress and HIV outcomes underscores the need to adopt methods for improving psychological wellness during these increasingly challenging times. We present empirically supported approaches that may assist providers in adopting resilience-focused HIV care as a strategy to respond to the increased need for psychological support in people with HIV during this time. We include recommendations for how providers might actualize this framework, though tailoring or adaptations will be necessary to fit contextual factors of each care setting, noting that country, culture, and political realities are also important considerations, including in low- and middle-income countries (26–28). Our definition of resilience-focused HIV care, built from a synthesis of recent HIV literature and assumptions of trauma-informed care, marks a novel contribution to the knowledge base and responds to the call for a multidimensional definition of resilience as part of HIV research. The key features of the proposed framework are conceptual and require testing. Although supported by the literature, these recommendations are not exhaustive and there may be other ways to fully adopt a resilience framework. Beyond the COVID-19 pandemic, systems of care having integrated a resilience framework will be better prepared for meeting the needs of persons with HIV during future disasters or adversity. We recommend that providers and organizations interested in integrating resilience-focused HIV care track progress and disseminate findings to advance this emerging field and provide lessons to others seeking to incorporate these practices.

## Data Availability Statement

The original contributions presented in the study are included in the article/supplementary material, further inquiries can be directed to the corresponding author/s.

## Author Contributions

LB conceptualized the manuscript, led the literature review, and drafted the work. EM, HK, HG, and BG contributed additional ideas and/or literature and revised the work critically for intellectual content. All authors contributed to the article and approved the submitted version.

## Author Disclaimer

The content is solely the responsibility of the authors and does not necessarily represent the official views of the National Institutes of Health.

## Conflict of Interest

The authors declare that the research was conducted in the absence of any commercial or financial relationships that could be construed as a potential conflict of interest.

## Publisher's Note

All claims expressed in this article are solely those of the authors and do not necessarily represent those of their affiliated organizations, or those of the publisher, the editors and the reviewers. Any product that may be evaluated in this article, or claim that may be made by its manufacturer, is not guaranteed or endorsed by the publisher.

## Footnotes

^1^Brown LL, Pennings J, Steckel S, Van Zyl M. *The Organizational Trauma Resilience Assessment: Methods and Psychometric Properties*. (Under Review).
